# Serine Hydroxymethyltransferase 1 Is Essential for Primary-Root Growth at Low-Sucrose Conditions

**DOI:** 10.3390/ijms23094540

**Published:** 2022-04-20

**Authors:** Yang Yuan, Danyun Xu, Denghao Xiang, Li Jiang, Honghong Hu

**Affiliations:** National Key Laboratory of Crop Genetic Improvement, College of Life Science and Technology, Huazhong Agricultural University, Wuhan 430070, China; yangyuan9109@163.com (Y.Y.); xudanyun2022@163.com (D.X.); dhxiang139@163.com (D.X.); jianglyy@163.com (L.J.)

**Keywords:** sucrose accumulation, root growth, ROS homeostasis, GSH, RBOHD

## Abstract

Plant roots are essential organs for absorbing nutrients from the soil or medium. Sucrose functions as a vital carbon source in root development, and sucrose starvation interferes with the redox state of plant cells. However, the mechanism of root growth at sucrose starvation remains unclear. Here, we report that SHMT1 (serine hydroxymethyltransferase 1) plays a crucial role in primary-root growth. *SHMT1* mutation caused decreased sugar levels, excessive H_2_O_2_ accumulation, and severe root-growth arrest at sucrose-free conditions, whereas plants with *SHMT1* overexpression had increased sugar and decreased H_2_O_2_ levels, and longer primary roots. Sucrose supply fully restored root growth of *shm1-2*, but CO_2_ alone could not, and SHMT1 is much more stable in roots than shoots at sucrose conditions, suggesting that SHMT1 accumulation in roots is critical for sucrose accumulation and root growth. Further ROS scavenging by GSH application or ROS synthesis inhibition by apocynin application or *RBOHD* mutation reduced H_2_O_2_ levels and partially restored the root-growth arrest phenotype of *shm1-2* at low-sucrose conditions, suggesting that SHMT1 modulates root growth via sucrose-mediated ROS accumulation. Our findings demonstrated the role of SHMT1 in primary-root growth by regulating sucrose accumulation and ROS homeostasis in roots.

## 1. Introduction

The plant roots are the first and direct organs that sense and respond to the rapid changes of the surrounding environment in the soil, in addition to absorbing nutrients and water from the soil [1]. Plant roots can be roughly divided into the meristem, elongation, and differentiation (maturation) zones [2]. The root length is mainly determined by the cell division within the meristem zone and the final cell length within the differentiation zone [3]. Plant hormones are essential for root development, such as auxin, cytokinin, abscisic acid, brassinosteroids, ethylene, and jasmonate [4]. Jasmonate suppresses cell division and elongation in roots [5], and ethylene enhances inhibition of root-cell elongation through promoting auxin biosynthesis and modulating auxin transport [6,7]. Unfavorable growth conditions, such as salt and osmotic stresses, also inhibit root growth and development mainly by disruption of auxin biosynthesis and redistribution of auxin in the root zones [8,9].

Carbon sources are also vital for root growth and development. Glucose functions as a signaling molecule in primary-root development, and the mechanisms are well-known [10,11]. Sucrose is a main product of photosynthesis, which plays an important role in energy supply during plant growth and development [12,13]. Recently, sucrose has also been proposed as a signal molecule to regulate the development of various plant organs, such as roots, leaves, stems, and flowers [14,15]. Exogenous application of sucrose can partially restore the root-growth inhibition caused by nutrient insufficiency or waterlogging, and induce the emergence of adventitious roots so as to better absorb nutrients from the soil [16,17]. Loss of function of *Arabidopsis* MEDIATOR (MED) complex altered auxin homeostasis and inhibited primary-root growth, and sucrose supply induced auxin-responsive gene expression to reactivate both cell division and elongation in primary roots [18]. Because sucrose can break down into glucose, fructose, and trehalose 6-phosphate (T6P), it is difficult to distinguish whether the effect of sucrose on root development is sucrose-specific or not. In *Arabidopsis*, sucrose increases the root length by enhancing root-meristem activity, and its effect is greater than that of equimolar glucose [19], suggesting that sucrose promotion of root growth is not only due to the hydrolysis of sucrose. Recent studies indicated that sucrose acts as a shoot-derived signaling molecule to trigger root morphogenesis in sunflower-seedling development [20], and as a cotyledon-derived long-distance signal to control root growth during early seedling development in Arabidopsis [21], supporting the view that transport of sucrose synthesized endogenously in shoots into the root tips is essential for root elongation, by integrating plant phytohormone-signaling pathways [21,22]. These studies suggest that sucrose plays a vital role in root growth. However, how plants regulate root growth, especially in sucrose-starvation conditions, is not clear.

Reactive oxygen species (ROS) are continuously produced during plant photosynthesis, respiration, and photorespiration, and play vital roles in plant growth and development, as well as in response to abiotic and biotic stresses [23]. Excessive quantities of ROS reduce the root-meristem size by inhibiting the expression of cell-cycle genes during cell division [24]. Among all kinds of ROS, hydrogen peroxide (H_2_O_2_) and superoxide (O_2_^−^) are two stable forms produced in chloroplasts, mitochondria, peroxisomes, and apoplasts of plant cells [25,26], and are essential for root growth [27,28,29,30]. O_2_^−^ accumulates in the meristem zone, while H_2_O_2_ mainly accumulates in the elongation zone [28]. Their spatial-distribution changes disturb the root-tip transition from a zone of proliferation to a zone of elongation and differentiation, thereby affecting root length [31].

The production of ROS is closely related to photosynthetic metabolites [32]. Soluble sugars not only regulate the photosynthetic activity via influencing the expression of photosynthesis-related factors, but also modulate ROS balance [33]. Excessive sugar accumulation or sugar starvation in plants leads to excessive ROS accumulation [33]. Glucose treatment reduces the levels of O_2_^−^ and H_2_O_2_ generated by abiotic stresses in plants [34]. Exogenous sucrose supply enhances the resistance of Arabidopsis seedlings to the herbicide atrazine by reducing the levels of singlet oxygen (^1^O_2_) and H_2_O_2_ [35,36]. Sucrose supply also alleviate NH_4_^+^ excess stress responses and ROS burst, which promote the assimilation and conversion of carbon and nitrogen, thereby promoting the growth of roots and shoots [37]. Additionally, sucrose starvation in plants induces the expression of ROS oxidative stress genes to produce excessive ROS, and increases the activities of ACX4 enzymes in oxidation of short-chain fatty acids and CAT3 enzymes to scavenge the over-accumulated H_2_O_2_ [38]. However, little is known about the specific relationship between sucrose starvation and ROS in modulating root growth.

SHMT1 encodes a key enzyme in the photorespiration process, which catalyzes the conversion of glycine into serine [39,40]. Mutation of *SHMT1* clearly exhibited chlorotic and lethal phenotypes under ambient growth conditions [41,42], which were rescued by elevated CO_2_ [43,44]. *SHMT1* is also reported to participate in biotic or abiotic stress responses [45,46]. In this study, we demonstrated the crucial role of *SHMT1* in primary-root growth via linking the sucrose with the redox state in *Arabidopsis*. *shm1-2* mutant exhibited a severe root-retardation phenotype at low-sucrose conditions with increased H_2_O_2_ levels, and plants with *SHMT1* overexpression had longer roots with reduced H_2_O_2_ levels. Further sugar-feeding assays, ROS scavenging by GSH, or reduction of *RBOHD* expression partially restored the root-growth inhibition phenotype at low-sucrose conditions (0%, 0.5% sucrose), demonstrating that *SHMT1* participates in primary-root growth by sucrose-modulated redox state.

## 2. Results

### 2.1. Mutation of SHMT1 Caused Reduced Primary-Root Growth under Sucrose-Free Conditions

**Figure 1:**
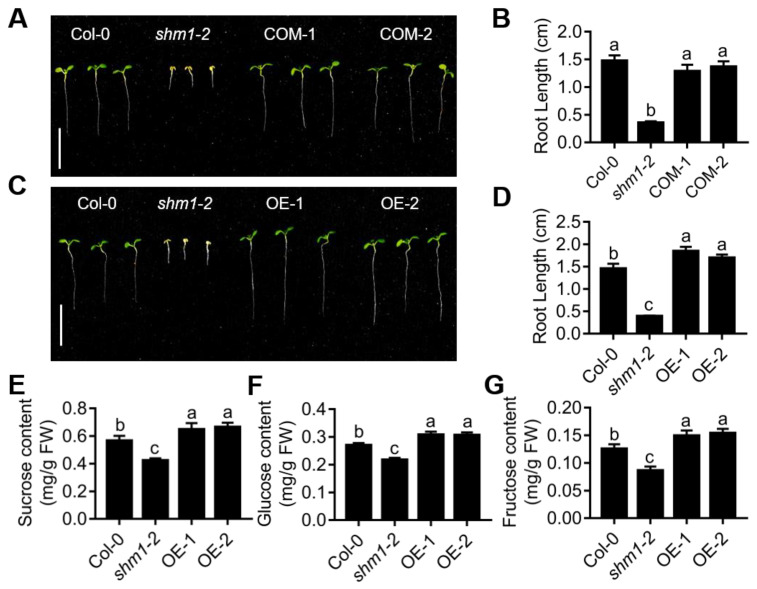
*SHMT1*-promoted primary-root growth at sucrose-free conditions. (**A**) The root-growth phenotype of Col-0, *shm1-2* and *SHMT1* complementation lines (COM) grown on sucrose-free 1/2 MS medium for 8 days. (**B**) The primary-root length was statistically analyzed, as shown in (**A**). (**C**,**D**) The root-growth phenotype (**C**) and primary-root length (**D**) of *SHMT1* overexpression lines (OE) grown on sucrose-free 1/2 MS medium for 8 days. Bars = 1 cm. Data presented were means ± SEM, *n* = 3 experiments; each experiment had 10 roots per genotype. (**E**–**G**) The contents of sucrose (**E**), glucose (**F**), and fructose (**G**) in Col-0, *shm1-2* and *SHMT1* OE seedlings grown on sucrose-free 1/2 MS medium for 8 days. Data presented were means ± SEM, *n* = 3 biological replicates. Different letters above error bars indicate significant difference at *p* < 0.05, using one-way ANOVA with Tukey’s test.

*SHMT1* is reported to be involved in abiotic and biotic stress responses, such as salt, drought, and ionic lead (Pb) stresses [45,46,47,48]. Our experiments and microarray data from other labs showed that *SHMT1* was induced by sucrose (Supplementary Figure S1), indicating that *SHMT1* may be also involved in sucrose-mediated plant development or responses. We then determined the root growth of *shm1-2* and Col-0 on 1/2 MS medium without sucrose for 8 days. The root length of *shm1-2* was extremely shorter than that of Col-0, and the *shm1-2* mutant had smaller and chlorotic cotyledons at sucrose-free conditions (Figure 1A,B), consistent with the previous studies where mutation of *SHMT1* exhibited smaller plant size in soil [40,49]. When the full-length CDS of *SHMT1* driven by the UBQ10 promoter was introduced into the *shm1-2* mutant, the primary-root-growth arrest and cotyledon chlorotic phenotypes of *shm1-2* were all rescued to the similar levels as Col-0 in the *SHMT1*-expression complementation lines (COM) (Figure 1A,B and Supplementary Figure S2A), suggesting that *SHMT1* is the causative gene for the root-growth retardation observed in *shm1-2* at sucrose-free conditions. To further explore the role of *SHMT1* in root growth, we overexpressed *SHMT1* in Col-0. Interestingly, the primary root length of *SHMT1* overexpression lines (OE) was longer than that of Col-0 (Figure 1C,D and Supplementary Figure S2B). These results revealed the correlation of *SHMT1* expression with primary-root length, and suggested that *SHMT1* was involved in the primary-root-growth regulation at sucrose-free growth conditions.

SHMT1 is an enzyme in the photorespiratory pathway, whose mutation causes decreased carbon recycling during photorespiration [42], and finally reduces photosynthesis and carbohydrate accumulation. We then determined the sucrose, glucose, and fructose contents in 8-day-old Col-0, *shm1-2* and *SHMT1* OE seedlings grown at sucrose-free conditions. The measurements showed that the levels of sucrose, glucose, and fructose were all significantly lower in the *shm1-2* mutant and greater in the *SHMT1* OE seedlings compared to Col-0 at sucrose-free conditions (Figure 1E–G). The primary root length positively correlated with these carbohydrate contents in these genotypes, indicating that the primary-root-growth differences in the *SHMT1* mutant or overexpression plants might be caused by different levels of these carbohydrates. Reduced levels led to shorter primary roots, and greater levels resulted in longer ones.

### 2.2. Exogenous Sucrose Rescued the Root-Growth-Arrest Phenotype of shm1-2 Mutant, but Elevated CO_2_ Alone Could Not

**Figure 2:**
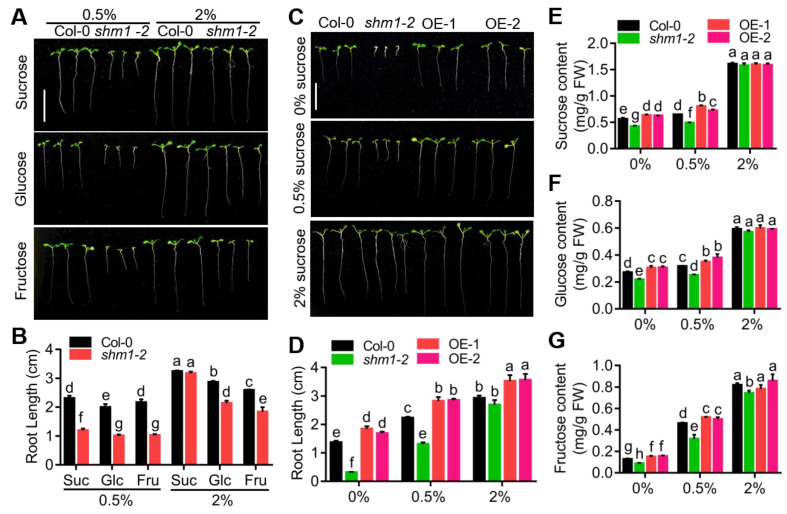
Exogenous sucrose supply rescued the root-growth-arrest phenotype of *shm1-2* mutant. (**A**,**B**) The root-growth phenotype (**A**) and root length (**B**) of *shm1-2* and Col-0 on 1/2 MS medium containing 0.5% or 2% sugars for 8 days. (**C**,**D**) The root-growth phenotype (**C**) and root length (**D**) of Col-0, *shm1-2* and *SHMT1* OE grown on 1/2 MS medium containing 0%, 0.5%, or 2% sucrose for 8 days. Bars = 1.5 cm. Data presented were means ± SEM, *n* = 3 experiments; each line had 10 roots per experiment. (**E**–**G**) The content of sucrose (**E**), glucose (**F**), and fructose (**G**) in Col-0, *shm1-2* and *SHMT1* OE seedlings in (**C**). Data presented were means ± SEM, *n* = 3. Different letters above error bars indicated significant difference at *p* < 0.05, using two-way ANOVA with Tukey’s test.

To determine that the root-growth arrest of the *shm1-2* mutant at sucrose-free conditions was mainly due to the deficiency of sucrose, glucose, or fructose, we grew Col-0 and *shm1-2* plants on the 1/2 MS medium supplemented with different concentrations of sucrose, glucose, or fructose. Sucrose supply promoted primary-root growth of both Col-0 and *shm1-2* plants, and the reduced *shm1-2* primary-root length was fully rescued by 2% sucrose (Figure 2A,B). Glucose or fructose supply only partially recovered the root-growth retardation of *shm1-2* compared to the effect of the same concentrations of sucrose (Figure 2A,B). The sucrose had greater effect than glucose and fructose on restoration of *shmt1-2* root growth, indicating that the shorter root length in *shm1-2* was not only caused by the deficiency of sugar for energy supply, and that sucrose may also act as a metabolite in root growth. The effects of exogenous sucrose on root growth were also tested in the *SHMT1* OE plants, which consistently showed longer primary-root length compared to Col-0 at all concentrations of sucrose conditions (Figure 2C,D). Additionally, the sucrose, glucose, and fructose contents were determined in the *shm1-2* and *SHMT1* OE seedlings supplemented with various concentrations of sucrose. Sucrose supply not only increased the sucrose level, but also the glucose and fructose levels in Col-0, *shm1-2* and *SHMT1* OE seedlings (Figure 2E–G), supporting the view that sucrose functions as a metabolite, which breaks down into glucose and fructose in plants. The levels of sucrose, glucose, and fructose were all significantly lower in the *shm1-2* mutant and greater in the *SHMT1* OE plants than those of Col-0 at low-sucrose conditions (Figure 2E–G); and at 2% sucrose conditions, Col-0, *shmt1-2*, and *SHMT1* OE had comparable levels of these three sugars. These feeding assays demonstrated that root-growth arrest in *shm1-2* was mainly caused by the lower level of sucrose, and that longer roots in *SHMT1* OE mainly resulted from the greater sucrose level at low-sucrose conditions. These results suggested that sucrose also acts as a signal molecule in *SHMT1*-mediated root growth in addition to being a metabolite for energy supply.

**Figure 3:**
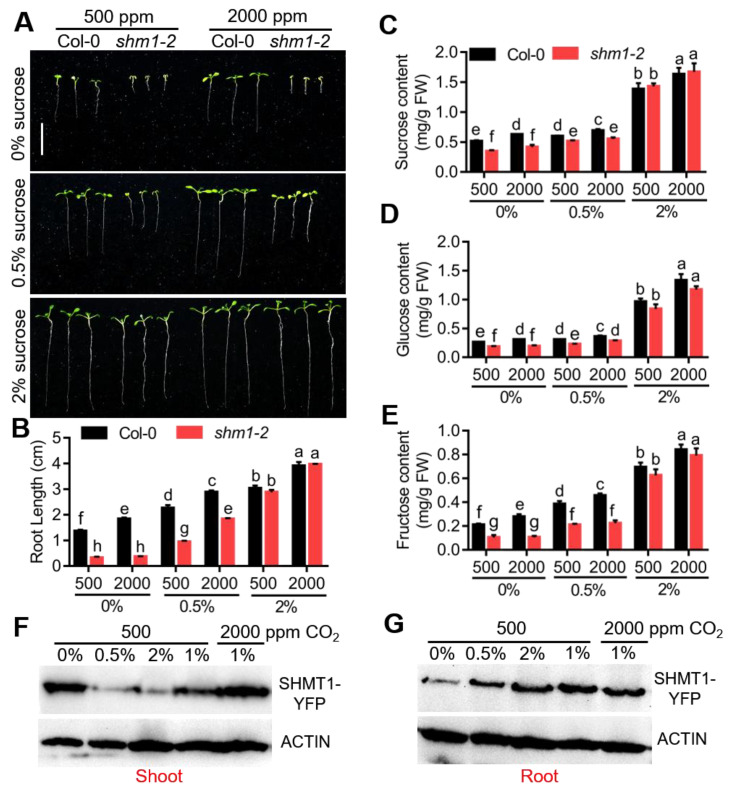
Elevated CO_2_ alone did not recover the growth-retardation phenotype of *shm1-2* at sucrose-free conditions. (**A**) The root-growth phenotype of *shm1-2* and Col-0 grown on 1/2 MS medium containing different concentrations of sucrose at 500 or 2000 ppm CO_2_ for 8 days. (**B**) The root length was statistically analyzed as shown in (**A**). Data presented were means ± SEM, *n* = 3 experiments, each with 10 roots per genotype. Bars = 1.5 cm. (**C**–**E**) The contents of sucrose (**C**), glucose (**D**), and fructose (**E**) in *shm1-2* and Col-0 seedlings grown on 1/2 MS medium containing 0%, 0.5%, or 2% sucrose at 500 or 2000 ppm CO_2_ for 8 days. Data presented were means ± SEM, *n* = 3 experiments. Different letters above error bars indicated significant difference at *p* < 0.05, using two-way ANOVA with Tukey’s test. Western blot analyses of SHMT1 protein levels in shoots (**F**) and roots (**G**) of 35S-SHMT1-YFP expressing transgenic plants grown on 1/2 MS medium containing 0%, 0.5%, 2% sucrose at 500 ppm CO_2_ or 1% sucrose at 500, 2000 ppm CO_2_ for 8 days. Actin was used as a loading control.

CO_2_ acts as a donor for carbohydrate synthesis, and high CO_2_ treatment represses photorespiration and increases sucrose synthesis in plants. A previous study reported that high CO_2_ concentrations could restore the growth arrest and chlorotic phenotypes of another *shmt1* mutant allele, *shm1-1*, grown in soil [44]. To test whether the reduced primary-root growth can be rescued by high CO_2_, *shm1-2* together with Col-0 was grown on 1/2 MS medium without sucrose at ambient (500 ppm) or elevated CO_2_ (2000 ppm) conditions. Elevated CO_2_ promoted primary-root growth of Col-0 at sucrose-free conditions; however, in the *shm1-2* plants, the primary-root growth was not accelerated by elevated CO_2_ (Figure 3A,B). We then also tested the effect of elevated CO_2_ on the primary-root growth of *shm1-2* at 0.5% or 2% sucrose conditions. Interestingly, the primary-root growth of both *shm1-2* and Col-0 was accelerated by elevated CO_2_ at 0.5% or 2% sucrose conditions compared to that under ambient CO_2_ conditions (Figure 3A,B). Moreover, elevated CO_2_ increased the levels of sucrose, glucose, and fructose in Col-0 compared to ambient CO_2_ conditions at all sucrose concentrations (Figure 3C–E). However, in *shm1-2*, elevated CO_2_ had no impact on sucrose and fructose accumulation at low-sucrose concentrations (0%, 0.5%) compared to ambient 500 ppm CO_2_ conditions. At 2% sucrose conditions, elevated CO_2_ further promoted sucrose, glucose, and fructose accumulation in *shm1-2* and there were no significant differences in the levels of these three sugars between Col-0 and *shm1-2* (Figure 3C–E), consistent with their primary-root growth (Figure 3A,B). These results suggested that *SHMT1* modulated primary-root elongation by affecting sucrose accumulation, and also indicated that during cotyledon development, a suitable level of sucrose, which might act as a signal, was required to activate early seedling development and photosynthesis.

To further explore the role of SHMT1 on modulation of primary-root growth, the expression level of *SHMT1* was detected in Col-0 shoots and roots grown on 1/2 MS medium with different concentrations of sucrose at ambient CO_2_ conditions (500 ppm) or with 1% sucrose at 500 and 2000 ppm CO_2_. *SHMT1* expression in shoots was increased by the increase in sucrose concentration; however, its expression in roots showed the opposite response. At 1% sucrose condition, *SHMT1* was suppressed by 2000 ppm CO_2_ either in shoots or in roots compared to 500 ppm CO_2_ (Supplementary Figure S3A,B). In addition, we also detected the SHMT1 protein levels in shoot and root tissues of *SHMT1-YFP* expressing transgenic plants, the protein levels gradually increased with the increasing sucrose concentration in roots, but in shoot tissues SHMT1 levels were significantly reduced at 0.5% and 2% sucrose conditions, indicating that SHMT1 is much more stable in roots at relatively high concentrations of sucrose. CO_2_ has no significant effect on SHMT1 protein levels, neither in roots nor shoots (Figure 3F,G). Together with the increase in root growth by sucrose addition and *SHMT1* overexpression (Figure 2C,D), these results implied that SHMT1 modulation of root growth mainly depended on its level in roots, and the role of SHMT1 in photorespiration contributed less to root growth.

### 2.3. Root-Meristem Activity in the shm1-2 Mutant Was Reduced at Low-Sucrose Conditions

**Figure 4:**
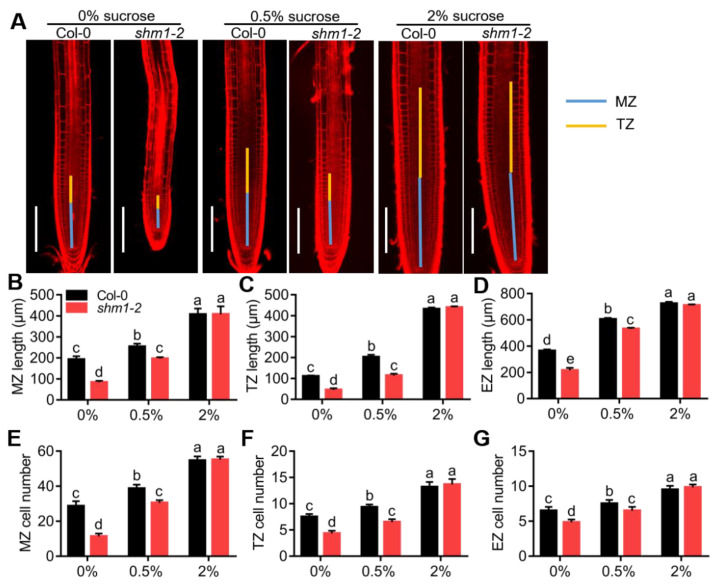
Root-meristem activity in the *shm1-2* mutant was reduced at low-sucrose conditions. (**A**) The root-meristem and transition-zone size of Col-0 and *shm1-2* at 0%, 0.5%, and 2% sucrose conditions. (**B**–**D**) The MZ, TZ, and EZ lengths of Col-0 and *shm1-2* at 0%, 0.5%, 2% sucrose conditions. (**E**–**G**) The corresponding cell number in the MZ, TZ and EZ at 0%, 0.5%, and 2% sucrose conditions. MZ, meristem zone, TZ, transition zone, EZ, elongation zone. Data presented were means ± SD, *n* = 3 experiments, each with 10 roots per experiment. Bars = 200 μm. Different letters above error bars indicated significant difference at *p* < 0.05, using two-way ANOVA with Tukey’s test.

We next measured root zone length, cell numbers, and cell length of MZ (meristem zone), TZ (transition zone), and EZ (elongation zone) in Col-0 and *shm1-2* roots at 0%, 0.5%, and 2% sucrose conditions. At sucrose-free conditions, *shm1-2* exhibited extremely shorter MZ, TZ, and EZ length, and fewer cell numbers in each zone than those of Col-0. When 0.5% sucrose was supplemented, the zone length and cell numbers of MZ, TZ, and EZ in Col-0 were significantly increased. Although the zone length and cell numbers in these three zones in *shm1-2* were still lower than those of Col-0 at 0.5% sucrose conditions, the difference was narrowed compared to the sucrose-free conditions. At 2% sucrose conditions, the zone length and cell numbers of MZ, TZ, and EZ in *shm1-2* were comparable with those of Col-0 (Figure 4). There were no significant differences in the cell length of these three zones between Col-0 and *shm1-2* at different sucrose conditions (Supplementary Figure S4A–C). These results indicated that *SHMT1* controlled root length at low-sucrose conditions by regulating cell division rather than cell elongation in these zones.

To further explore whether the reduced cell number in the *shm1-2* mutant was caused by the reduced cell-division activity, the expression levels of cell-cycle marker genes, *CYCB1;1* (AT4G37490), *CYCB1;2* (AT5G06150), and *CYCB3;1* (AT1G16330) [50,51], were detected. The expression levels of these three genes were significantly downregulated in the *shm1-2* mutant compared to Col-0 at low-sucrose conditions; 2% sucrose supply promoted the expression of these three genes and led to comparable levels in *shm1-2* and Col-0 (Supplementary Figure S4D), consistent with the cell numbers and root-length phenotypes at different concentrations of sucrose conditions (Figure 2 and Figure 4). These results implied that the inhibition of cell-division activity in the *shm1-2* mutant at low-sucrose conditions were caused by the reduced expression levels of cell-cycle genes.

### 2.4. Accumulation of H_2_O_2_ in the Primary Roots Was Altered by SHMT1 Expression Changes at Low-Sucrose Conditions

**Figure 5:**
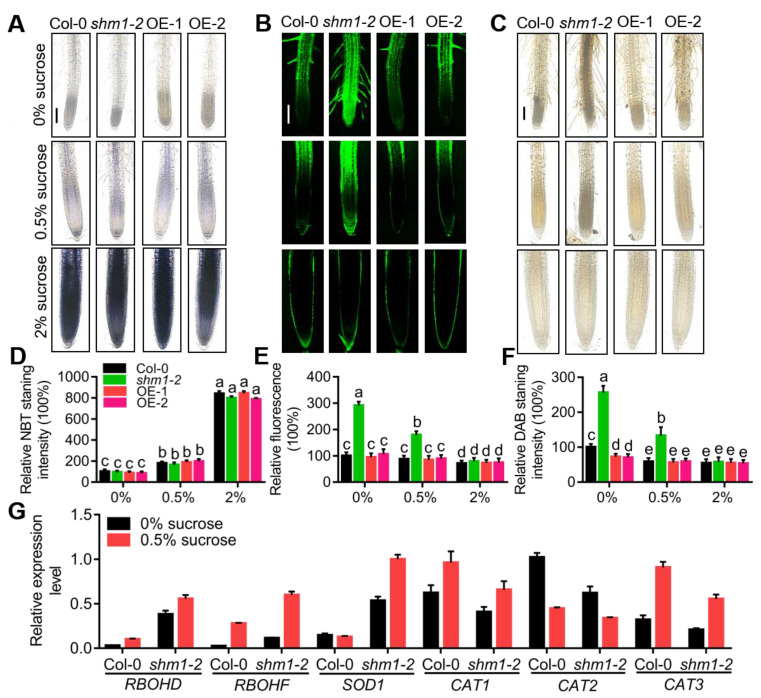
*SHMT1*-expression changes altered H_2_O_2_ accumulation in roots at low-sucrose conditions. (**A**–**C**) NBT staining for superoxide (O_2_^−^) (**A**), H_2_DCFDA (**B**) and DAB staining (**C**) for H_2_O_2_ in the primary roots of 8-day-old Col-0, *shm1-2* and *SHMT1* overexpression lines at 0%, 0.5%, and 2% sucrose conditions. Bars = 200 μm. (**D**–**F**) The NBT-staining intensity (**D**), fluorescence intensity (**E**), and DAB-staining (**F**) intensity in Col-0, *shm1-2*, OE-1 and OE-2 at 0, 0.5%, and 2% sucrose conditions by ImageJ in (**A**–**C**), respectively. The intensity in Col-0 roots at sucrose-free conditions was set as 100%. In (**D**–**F**), data presented were means ± SEM, *n* = 3 experiments, each with 10 roots per experiment. Different letters above error bars indicated significant difference at *p* < 0.05, using two-way ANOVA with Tukey’s test. (**G**) Quantitative PCR analyses of H_2_O_2_-producing genes (*RBOHD*, *RBOHF*, *SOD1*) and scavenging genes (*CAT1*, *CAT2*, *CAT3*) in 8-day-old seedlings of *shm1-2* and Col-0 at 0%, 0.5% sucrose conditions. Data presented were means ± SEM, *n* = 3.

Sucrose starvation/limitation in plants disturbs the oxidative state, thereby causing excessive accumulation of ROS [38]. To determine whether ROS accumulation was altered in the *shm1-2* mutant or *SHMT1* OE plants, the levels of superoxide (O_2_^−^, by NBT staining) and H_2_O_2_ (by H_2_DCF-DA and DAB staining) in roots were detected at 0%, 0.5%, and 2% sucrose conditions. With the increase in sucrose concentration, the accumulation of O_2_^−^ in the roots of Col-0, *shm1-2* and *SHMT1* OE increased greatly, and had no significant differences among these genotypes (Figure 5A,D). The *shm1-2* mutant obviously accumulated more H_2_O_2_ than Col-0 at low-sucrose conditions, as indicated by H_2_DCF-DA and DAB staining, which was reduced to the similar level as Col-0 at 2% sucrose conditions. Moreover, the *SHMT1* OE roots had reduced H_2_O_2_ levels (DAB staining) (Figure 5B,C,E,F), in accordance with their primary-root-length phenotypes at low-sucrose conditions (Figure 2C,D). These results suggested that change of *SHMT1* expression influenced the accumulation of H_2_O_2_, but not superoxide, in roots at low-sucrose conditions, and *SHMT1* modulated root length possibly through regulation of H_2_O_2_ levels.

The homeostasis of ROS in plants is balanced by production and scavenging [52]. Oxygen (O_2_) is reduced by NADPH oxidase to superoxide (O_2_^−^) using NADPH as an electron donor [53]. The superoxide is released into the apoplastic space and dismutated into H_2_O_2_ by superoxide dismutase (SOD) [54]. Peroxidases facilitates the conversion of H_2_O_2_ into H_2_O and O_2_ [55]. To determine whether H_2_O_2_ overaccumulation in the *shm1-2* mutant was due to the defect of H_2_O_2_ production or scavenging, the expression levels of NADPH oxidase genes (*RBOHD/F*, *Respiratory Burst Oxidase Homologue D/F*), *SOD1*, and catalase genes (*CAT1*, *CAT2* and *CAT3*) were detected in Col-0 and *shm1-2* seedlings grown on 1/2 MS medium with 0% and 0.5% sucrose. Expression levels of *RBOHD*, *RBOHF*, and *SOD1* were significantly upregulated in *shm1-2* compared to Col-0 at low-sucrose conditions, and those of *CAT1*, *CAT2*, and *CAT3* were obviously downregulated (Figure 5G). The activities of SOD (which catalyzes superoxide into H_2_O_2_) and CAT (which scavenges H_2_O_2_) were measured in the 8-day-old seedlings of Col-0 and *shm1-2* supplied with different concentrations of sucrose. SOD activity was greatly increased and CAT activity was significantly decreased in *shm1-2* compared to Col-0 at low-sucrose conditions (0%, 0.5%) (Supplementary Figure S5A), while at 2% sucrose conditions, *shm1-2* and Col-0 had comparable SOD and CAT activities (Supplementary Figure S5). These results were consistent with the expression levels of H_2_O_2_-producing and scavenging genes (Figure 5G), and suggested that the increased H_2_O_2_ accumulation in the *shm1-2* mutant was caused by the increased H_2_O_2_ production and reduced H_2_O_2_ scavenging. Taken together, our results revealed that *SHMT1*-expression-level changes influenced H_2_O_2_ accumulation in the primary roots and indicated that the inhibition of primary-root growth in *shm1-2* at low-sucrose conditions was possibly due to the high oxidative status caused by H_2_O_2_ overaccumulation.

### 2.5. GSH Treatment Partially Rescued the Reduced Root Growth of shm1-2 during Sucrose Starvation

**Figure 6:**
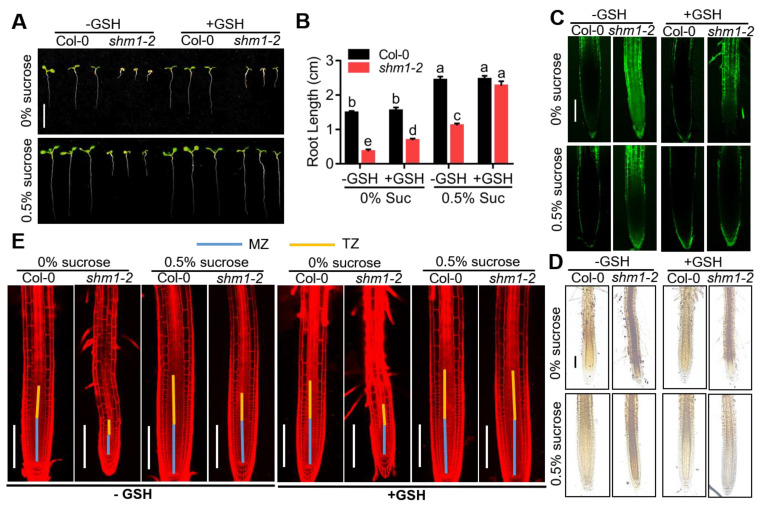
GSH treatment partially rescued the growth-arrest phenotype of the *shm1-2* mutant at low-sucrose conditions. (**A**) The primary-root-growth phenotype of Col-0 and *shm1-2* at low-sucrose conditions plus 200 μM GSH or not for 8 days. Bars = 1 cm. (**B**) Statistical analyses of the root length in (**A**). Different letters above error bars indicated significant difference at *p* < 0.05, using two-way ANOVA with Tukey’s test. (**C**,**D**) H_2_DCFDA (**C**) and DAB (**D**) staining for H_2_O_2_ in primary roots of 8-day-old Col-0 and *shm1-2* seedlings at low-sucrose conditions plus 200 μM GSH or not. Bars = 200 μm. (**E**) The root-meristem and transition-zone size of Col-0 and *shm1-2* at low-sucrose conditions plus 200 μM GSH or not for 8 days. Bars = 200 μm.

Previous studies reported that H_2_O_2_ could be scavenged by GSH antioxidant [30,56]. To further explore whether H_2_O_2_ scavenging could restore cell division of root tips in *shm1-2*, the plants were grown on the 1/2 MS medium with low sucrose plus 200 μM GSH or not for 8 days. As expected, GSH treatment greatly rescued the primary-root-growth retardation of *shm1-2* at low-sucrose conditions (Figure 6A,B). H_2_O_2_-level determination of these roots showed that application of GSH greatly reduced the accumulation of H_2_O_2_ in *shm1-2* roots at sucrose-free conditions, and reduced to the similar level as Col-0 at 0.5% sucrose conditions (Figure 6C,D and Supplementary Figure S6A,B), suggesting that GSH was able to scavenge H_2_O_2_ in the roots and H_2_O_2_ accumulation was a major cause of primary-root arrest in *shm1-2* at low-sucrose conditions. The zone length and cell numbers in MZ, TZ, and EZ of these roots at low-sucrose conditions with or without 200 μM GSH were also analyzed. The length and cell numbers of MZ, TZ, and EZ zones in *shm1-2* were greatly rescued at sucrose-free conditions, and completely restored at 0.5% sucrose conditions by GSH application (Figure 6E and Supplementary Figure S6C–H). Excessive accumulation of ROS inhibits the expression levels of cell-cycle genes [30]. In the *shm1-2* plants, the cell-cycle genes (*CYCB1;1* and *CYCB1;2*) were significantly decreased at low-sucrose conditions (Supplementary Figure S4); we then detected the expression levels of cell-cycle genes *CYCB1;1* and *CYCB1;2* in these GSH treated or untreated seedlings. qPCR analyses showed that GSH application increased the levels of these cell-cycle genes in both Col-0 and *shm1-2*, and the expression levels in the mutant were restored to the similar levels as in Col-0 at 0.5% sucrose conditions (Supplementary Figure S7). These results illustrated that GSH treatment reduced the H_2_O_2_ level in the *shm1-2* roots, which then activated the cell-cycle gene expression and alleviated the arrest of root growth during sucrose starvation.

### 2.6. Mutation of RBOHD Partially Restored the Root-Growth Arrest of shm1-2 during Sucrose Starvation

**Figure 7:**
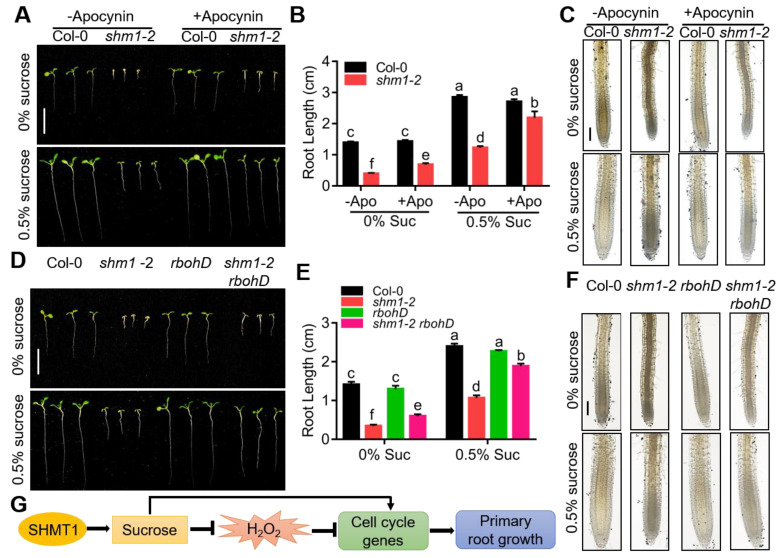
Application of NADPH oxidase inhibitor or mutation of *RBOHD* partially restored the root-arrest phenotype of *shm1-2* at low-sucrose conditions. (**A**) The primary-root-growth phenotype of Col-0 and *shm1-2* at low-sucrose conditions plus 10 μM apocynin (NADPH oxidase inhibitor) or not for 8 days. Bars = 1 cm. (**B**) Primary-root length was statistically analyzed, as shown in (**A**). (**C**) DAB staining for H_2_O_2_ in the primary roots of 8-day-old Col-0 and *shm1-2* at low-sucrose conditions plus 10 μM apocynin or not. Bars = 200 μm. (**D**,**E**) The primary-root-growth morphology (**D**) and root length (**E**) of Col-0, *shm1-2*, *rbohD* and *shm1-2 rbohD* at low-sucrose conditions for 8 days. Bars = 1 cm. (**F**) DAB staining for H_2_O_2_ in primary roots of 8-day-old Col-0, *shm1-2*, *rbohD*, and *shm1-2 rbohD* at low-sucrose conditions. Different letters above error bars indicate significant differences at *p* < 0.05, using two-way ANOVA with Tukey’s test. Bars = 200 μm. (**G**) A proposed model for *SHMT1* in primary-root-growth regulation. SHMT1 in roots promotes the accumulation of sucrose to inhibit H_2_O_2_ accumulation; the reduced H_2_O_2_ level then activates expression of cell-cycle genes and increases root-meristem activity, thereby promoting primary-root growth. The increased sucrose may also directly increase the expression of cell-cycle genes to promote primary-root growth.

H_2_O_2_ accumulation in *shm1-2* was caused by H_2_O_2_ overproducing and less scavenging, and NADPH oxidase genes *RBOHD* and *RBOHF* were dramatically upregulated in *shm1-2* at low-sucrose conditions (Figure 5G). To confirm that H_2_O_2_ overaccumulation in the *shm1-2* mutant was caused by the increase of NADPH oxidase activity at sucrose-limited conditions, Col-0 and *shm1-2* seedlings treated with 10 μM apocynin (NADPH oxidase inhibitor) were used to observe the root growth and H_2_O_2_ level at low-sucrose conditions. Apparently, reducing the activity of NADPH oxidase partially restored the root-growth-arrested phenotype in *shm1-2* at low-sucrose conditions, especially at 0.5% sucrose (Figure 7A,B). Moreover, the H_2_O_2_ level in *shm1-2* detected by DAB staining was significantly reduced after apocynin treatment at low-sucrose conditions (Figure 7C and Supplementary Figure S8A). The expression level of *RBOHD* was increased more than *RBOHF* in *shm1-2* (Figure 5G) and previous studies reported that mutation of *RBOHD* caused decreased H_2_O_2_ levels [57,58]. The *shm1-2* and *rbohD* (CS9555) single mutants were then crossed to generate the double mutant. As expected, the root length of the *shm1-2 rbohD* double mutant was obviously longer than that of the *shm1-2* single mutant at low-sucrose conditions, especially at 0.5% sucrose conditions (Figure 7D,E). Moreover, the mutation of *RBOHD* in *shm1-2* dramatically decreased the H_2_O_2_ level in roots at low-sucrose conditions (Figure 7F and Supplementary Figure S8B). These results revealed that decreasing the H_2_O_2_ level by reducing NADPH oxidase activity, which was caused by *RBOHD* mutation, could partially restore the root retardation in *shm1-2* at low-sucrose conditions, further suggesting the important role of ROS accumulation in regulation of root growth during sucrose starvation.

## 3. Discussion

Sucrose, a main product of photosynthesis, controls various developmental and metabolic processes in plants [14]. Sucrose starvation causes enormous changes in gene expression, enzyme activity, and cellular morphology [59]. However, the mechanisms underlying the sucrose deficiency on plant-root elongation still remain unclear. Here, we demonstrate that Arabidopsis *SHMT1*, which encodes a key enzyme in the photorespiratory pathway with mitochondrial serine hydroxymethyltransferase activity, is essential for primary-root growth at low-sucrose conditions.

*SHMT1* mutation caused extremely short roots (Figure 1A,B), and *SHMT1* overexpression resulted in longer roots in the sucrose-free growth medium (Figure 1C,D). The correlation of *SHMT1* expression level and primary-root growth at sucrose-free conditions demonstrated the involvement of SHMT1 in root growth during sucrose starvation. Sugar measurements (Figure 1E–G) and feeding assays (Figure 2A,B) suggested that the alterations of root growth were due to the sugar-level differences. Sucrose supply had greater promotion on *shm1-2* root growth than the same concentration of other sugars did (Figure 2A,B), such as glucose and fructose, suggesting that sucrose plays a more dominant role than other sugars in *SHMT1*-mediated primary-root growth. Moreover, sucrose can break down into fructose and glucose, and sucrose supply increased glucose and fructose levels in *shm1-2* (Figure 2E–G). These results suggest that *SHMT1* regulates primary-root growth mainly through modulation of sucrose accumulation, and sucrose not only acts as a metabolite, but also as a signal molecule in primary-root growth mediated by *SHMT1*.

CO_2_ is an indispensable source for plant photosynthesis to synthesize carbohydrates, and elevated CO_2_ is widely reported to promote plant photosynthesis and suppress the photorespiration process [60,61]. Elevated CO_2_ (2000 ppm) enhanced the primary-root elongation of Col-0 compared to the ambient CO_2_ (500 ppm) at various concentrations of sucrose conditions; however, its effect on *shm1-2* root growth at sucrose-free conditions was abolished (Figure 3A,B). These results implied that a minimum level of sucrose stored in seeds was required to ensure early seedling development and normal photosynthesis. SHMT1 protein was more stable in roots and unstable in shoots at sucrose conditions (Figure 3F,G), and *SHMT1* OE plants had longer roots at sucrose-free conditions (Figure 2C,D), suggesting that the SHMT1 level in roots directly modulates sucrose accumulation, which is critical for primary-root growth. Sucrose supply decreased SHMT1 protein levels, and high CO_2_ suppressed *SHMT1* expression in shoots, suggesting that photorespiration contributes less in SHMT1-mediated primary-root growth. *SHMT1* mutation caused sucrose accumulation lower than this minimum level, inhibiting early seedling development and primary-root growth. The measurement of sugar contents at these conditions supported this view (Figure 3C–E). The previous studies reported that the growth-arrest phenotype of *shm1-1* grown in soil could be well-restored at high-CO_2_ conditions [43,44], which could be explained by the organic fertilizers and microorganisms in the soil providing carbon sources, thus promoting root growth and seedling development. All these results suggested that *SHMT1* modulated primary-root growth through positively regulating sucrose accumulation in roots. We speculated that in addition to being an enzyme in the photorespiration pathway, SHMT1 may also have a role in regulating the stability of sucrose synthesis-related proteins, or affecting the enzymatic activity of sucrose synthase, thus influencing sucrose accumulation in roots.

Sucrose starvation causes degradation of key cell-cycle proteins, such as CYCD3;1 and RBR1 in a proteasome-dependent manner [62,63] to inhibit cell proliferation during development in *Arabidopsis*. Our results showed that at low-sucrose conditions, SHMT1 modulated root growth mainly via regulating cell division by influencing the expression levels of key cell-cycle genes in roots. The zone length and cell numbers in each root zone were consistent with the expression levels of *CYCB1;1*, *CYCB1;2*, and *CYCB3;1* in *shm1-2* at low-sucrose conditions (Figure 4 and Supplementary Figure S4), suggesting that sucrose starvation led to the reduced expression of these cell-cycle genes, thereby causing the inhibition of root-meristem activity.

It is well known that excessive ROS inhibit the root elongation through suppressing the cell division [64,65,66]. Previous studies have shown that sucrose starvation disturbs ROS status. For example, H_2_O_2_ is produced more as a by-product in the roots of autophagy-related mutants [67], and CAT3 activity is increased to scavenge the over-accumulated H_2_O_2_ in *Arabidopsis* at sucrose-starvation conditions [38]. In this study, we found that ROS—mainly H_2_O_2_—were over-accumulated in the *shm1-2* mutant during sucrose starvation, which was caused by the increased H_2_O_2_ production and reduced scavenging process (reduced CAT activity) (Figure 5 and Supplementary Figure S5). The supplement of GSH or NADPH oxidase inhibitor in *shm1-2* greatly reduced ROS accumulation, restored the reduced expression of cell-cycle genes, rescued the root-meristem activity and cell division, and partially recovered the root-growth retardation at low-sucrose conditions (Figure 6, Figure 7 and Supplementary Figures S6–S8A), providing several lines of evidence that the short root length was caused by overaccumulated ROS. Moreover, mutation of *RBOHD* in *shm1-2* also reduced the ROS level and partially restored the root-growth-inhibition phenotype of *shm1-2* at low-sucrose conditions (Figure 7D–F and Supplementary Figure S8B). These results at pharmacological and genetic levels demonstrated that the primary-root-growth retardation in *shm1-2* mutant during sucrose starvation is directly linked to the redox state of root cells.

We proposed a model for SHMT1 in primary-root-growth regulation. Under low-sucrose conditions, SHMT1 promotes sucrose accumulation in roots, which inhibits H_2_O_2_ accumulation in roots. The reduced H_2_O_2_ level activates expression of cell-cycle genes and increases root-meristem activity, thereby promoting primary-root growth. The increased sucrose level may also directly increase the expression of cell-cycle genes to promote primary-root growth (Figure 7G). Our study reveals that *SHMT1* is involved in primary-root growth through modulation of sucrose accumulation and ROS status. Furthermore, our findings also shed light on the role of sucrose and ROS in primary-root growth. Moreover, our study also suggests a potential role of SHMT1 in improvement of plant tolerance to drought stresses and survival in barren soil by increasing root length.

## 4. Materials and Methods

### 4.1. Plant Materials and Growth Conditions

All Arabidopsis lines used in this study were in the Columbia background (Col-0). The mutant line *shm1-2* (SALK_083735, a T-DNA insertion mutant that insert in the last exon of *SHMT1*) was obtained from Dr. Yongfei Wang (Shanghai Institute of Plant Biotechnology, Shanghai, China) and *rbohD* was kindly provided by Dr. Shunping Yan (Huazhong Agricultural University, Wuhan, China). Plants were grown on half Murashige and Skoog (1/2 MS) medium containing different supplements in CO_2_-controlled growth chambers (Percival), in which CO_2_ concentrations can be accurately and well-controlled in a range of 100–2000 ppm with controlled conditions (22 °C, 16 h light/8 h night regime, 100 μmol photons m^−2^ s^−1^ light intensity, and 70% relative humidity).

### 4.2. Phenotype Identification and Root-Length Measurement

Seeds were surface-sterilized and grown on vertical 1/2 MS plates with different concentrations of sugars (0%, 0.5%, 2% *m*/*v*) for 8 days. Roots were imaged with a Nikon camera and the root length was measured with ImageJ software

### 4.3. Generation of SHMT1 Complementation and Overexpression Transgenic Plants

The coding sequence (CDS) of *SHMT1* was amplified by the primer pairs listed in Supplementary Table S1. The purified PCR product was digested with *Bam*HI and *Spe*I, and cloned into the corresponding sites of the BarII-UBQ10-MCS vector (Tang et al., 2020). The *UBQ10:SHMT1* construct was transformed into *shm1-2* and Col-0 via the floral-dipping method [68] to generate the complementation and overexpression plants, respectively. To generate the *35S:SHMT1-YFP*, the full length CDS of *SHMT1* was amplified and recombined into the vectors of pEarleygate101, then the *35S:SHMT1-YFP* construct was transformed into Col-0 to obtain *SHMT1-YFP* overexpression plants. The transgenic plants were all screened on soil with basta spraying.

### 4.4. Semiquantitative PCR and Quantitative Real-Time PCR

Total RNA was extracted from whole seedlings using TRIzol reagent (Invitrogen Life Technologies, Carlsbad, CA, USA), according to the manufacturer’s instructions. The cDNA was reverse-transcribed using M-MLV reverse transcriptase (Promega, Madison, WI, USA) according to the provided protocols. *Actin7* was used as an internal control for RT-PCR. Quantitative RT-PCR was performed by using a Universal SYBR^®®^ Green kit and the C1000 Touch Thermal Cycler real-time PCR detection system (Bio-Rad, Hercules, CA, USA). The *EF1α* gene was used as a reference for qRT-PCR. The primers used are listed in Supplementary Table S1.

### 4.5. Measurements of Sucrose, Glucose, and Fructose Contents

Sucrose, glucose, and fructose were extracted from 8-day-old seedlings and their contents were measured using Colorimetric/Spectrophotometric Assay kits (Comin Biotechnology Co., Ltd., Suzhou, China) for these three sugars as described [69]. Absorbances were measured at 340 nm (A340) by a Mithras LB 940 microplate analyzer.

### 4.6. Western Blot Analyses

Proteins were extracted from 10-day-old *35S-SHMT1-YFP* expressing transgenic plant roots. Crude extracts were centrifuged at 12,000 rpm for 10 min at 4 °C and supernatant proteins separated by 10% SDS-PAGE gel. The proteins were transferred onto a PVDF membrane. Membranes were blocked in 5% skim-milk TBS-T buffer for 1 h, and then incubated with antibodies against GFP (ABclonal, Wuhan, China) overnight at 4 °C. The membranes were then washed three times with TBS-T buffer, and incubated with diluted 1:10,000 antimouse HRP secondary antibodies for 2 h at room temperature. The detection was performed by Thermo Scientific Pierce ECL Kit (Thermo Fisher Scientific, Waltham, MA, USA).

### 4.7. Analyses of Zone Length, Cell Numbers, and Cell Length in Root Tips

Seedlings were grown vertically on 0%, 0.5%, or 2% sucrose containing 1/2 MS medium for 8 days, supplemented with or without 200 μmol GSH. Roots were incubated in 10 μmol propidium iodide (Sigma-Aldrich, St. Louis, USA) for 3 min, which was used to visualize the root cells, and were then imaged with a Leica TCS SP8 confocal microscope. The zone length, cell numbers, and cell length of meristem zone (MZ), transition zone (TZ), and elongation zone (EZ) in the cortex were measured and analyzed by ImageJ software as described [30]. The experiments were repeated at least three times, and in each measurement at least 10 roots per genotype were used.

### 4.8. Determination of ROS Levels

H_2_DCF-DA (2′,7′-dichlorodihydrofluoresin diacetate) (Sigma-Aldrich) and DAB (3′,3′-diaminobenzidine) were used for H_2_O_2_-level detection in roots as described [30]. Briefly, for H_2_DCF-DA staining, the roots of 8-day-old seedlings grown on 0%, 0.5%, or 2% sucrose containing 1/2 MS medium were immersed in 20 μmol H_2_DCF-DA buffer for 10 min and then washed by water. The fluorescence was determined by a laser scanning confocal (TCS SP8, Leica, Weztlar, Germany) microscope with excitation at 488 nm and emission at 525 nm. The relative fluorescence was analyzed, and the fluorescence in Col-0 roots without sucrose was set as 100%. DAB staining (1 mg/mL) and NBT staining (nitroblue tetrazolium, 1 mg/mL) for superoxide were performed according to the published methods [30]. The roots of 8-day-old seedlings grown on 0%, 0.5%, or 2% sucrose containing 1/2 MS medium plus 200 μmol GSH or 10 μmol apocynin or not were used.

### 4.9. Analyses of Antioxidant Enzyme

The 8-day-old seedlings grown on 0%, 0.5%, or 2% sucrose 1/2 MS medium were harvested and total proteins were extracted at 4 °C. Superoxide dismutase (SOD) and catalase (CAT) activities were measured with SOD and CAT assay kits (Comin Biotechnology Co., Ltd., Suzhou, China) as described [30]. A spectrophotometer (DU730, Beckman Coulter, Pasadena, CA, USA) was used to measure the absorbances at 560 nm and 240 nm for SOD and CAT, respectively.

### 4.10. Statistical Analysis

The data were statistically analyzed. Differences among different treatments or genotypes were assessed by one-way or two-way ANOVA with Turkey’s test. The *p* values less than 0.05 were considered statistically significant.

## Data Availability

No large datasets were generated as part of this study.

## Supplementary Materials

The following supporting information can be downloaded at: https://www.mdpi.com/article/10.3390/ijms23094540/s1.
