# Supplementary material for: Serine Hydroxymethyltransferase 1 Is Essential for Primary-Root Growth at Low-Sucrose Conditions
Source: Int J Mol Sci. 2022 Apr 20;23(9):4540. doi: 10.3390/ijms23094540 (PMC9100158; doi:10.3390/ijms23094540)
Supplement: Supplementary file 1 [file ijms-23-04540-s001.zip › ijms-1658072-supplementary.pdf]

Serine hydroxymethyltransferase 1 is essential for primary root growth at low sucrose conditions

Yang Yuan, Danyun Xu, Denghao Xiang, Li Jiang and Honghong Hu*

Supplemental Files

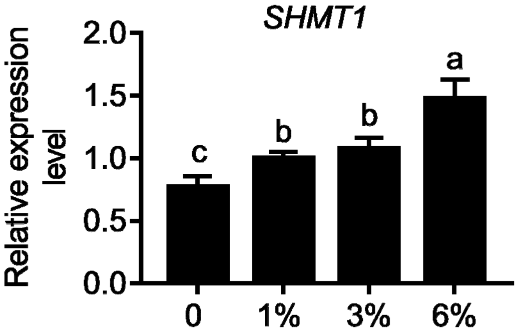

Supplemental Figure S1. Sucrose induced the expression level of *SHMT1*. Quantitative PCR analyses of *SHMT1* in Col-0 seedlings treated with different concentrations of sucrose for 6 h. Data presented were means ± SEM, n = 3. *EF1α* was used as an internal control.

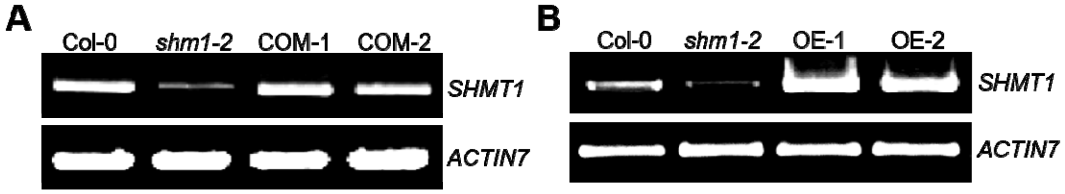

Supplemental Figure S2. Expression level of *SHMT1* in *shm1-2*, *SHMT1* complementation and overexpression lines. (**A**, **B**) The expression level of *SHMT1* in Col-0, *shm1-2*, *SHMT1* complementation lines (COM-1 and COM-2) (**A**) and overexpression lines (OE-1 and OE-2) (**B**). *ACTIN7* was used as an internal control.

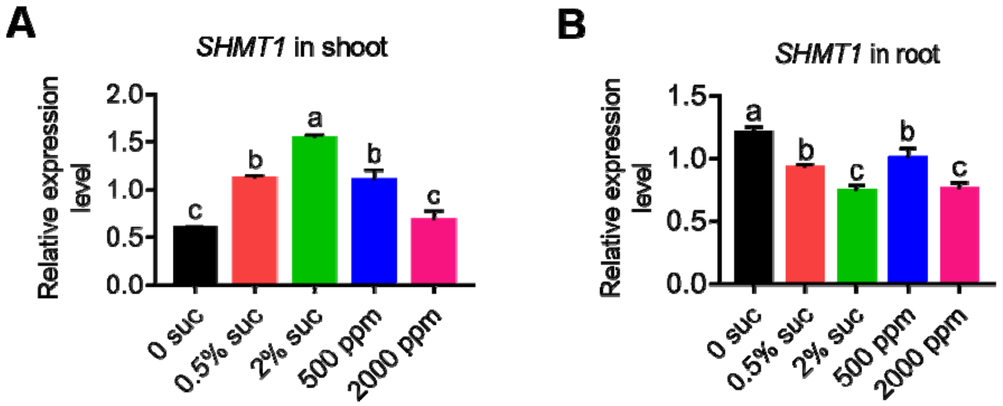

Supplemental Figure S3. Relative expression level of *SHMT1* in shoots and roots at different concentration of sucrose or CO_2 conditions. Quantitative PCR analyses of *SHMT1* in Col-0 shoots **(A)** and roots **(B)** grown on 1/2 MS medium containing 0, 0.5, 2% sucrose at 500 ppm CO_2 or 1% sucrose at 500, 2000 ppm CO_2 for 8 days. Data presented were means ± SEM, n = 3. *EF1α* was used as an internal control. Different letters above error bars indicated significant difference at $P < 0.05$.

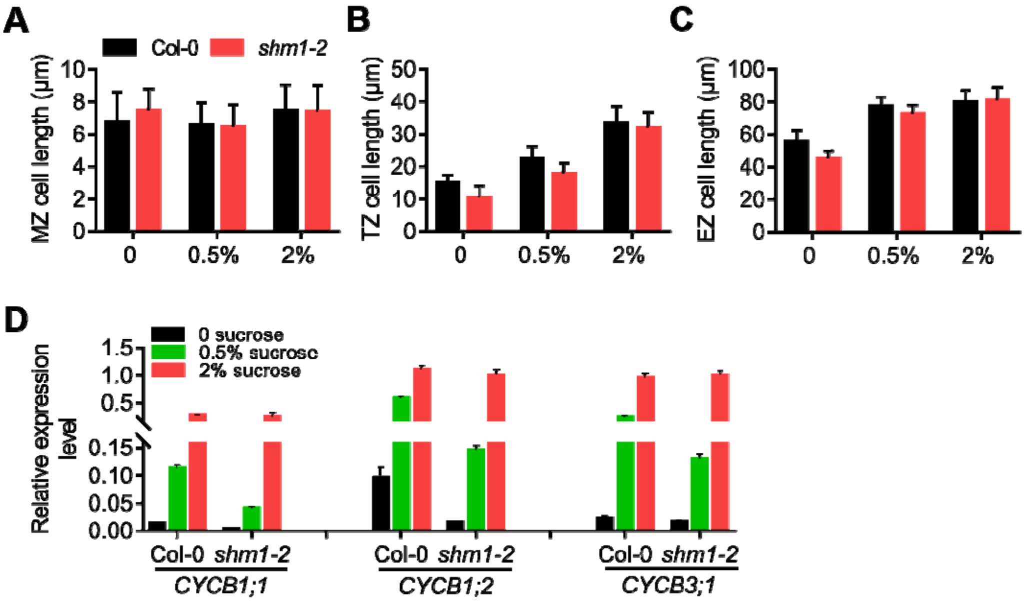

Supplemental Figure S4. The *shm1-2* mutant showed reduced root meristem activity at low sucrose conditions. (**A**-**C**) The corresponding cell length of Col-0 and *shm1-2* in the MZ (**A**), TZ (**B**), and EZ (**C**) at 0, 0.5%, and 2% sucrose conditions. MZ, meristem zone, TZ, transition zone, EZ, elongation zone. Data presented were means ± SD, *n* = 3 experiments, each with 10 roots per experiment. (**D**) Quantitative PCR analyses of cell cycle marker genes (*CYCB1;1*, *CYCB1;2*, and *CYCB3;1*) in *shm1-2* and Col-0 seedlings grown on 1/2 MS medium containing 0, 0.5%, or 2% sucrose. Data presented were means ± SEM, *n* = 3. *EF1α* was used as an internal control.

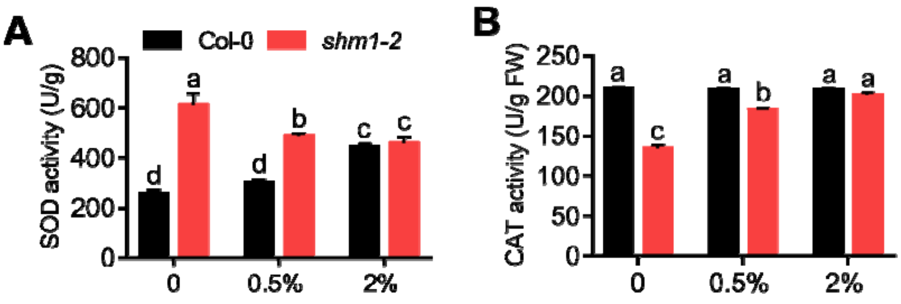

Supplemental Figure S5. SOD and CAT activities in the *shm1-2* mutant at different sucrose conditions. (**A**, **B**) Activities of superoxide dismutase (SOD) (**A**) and catalase (CAT) (**B**) in 8-day-old seedlings of Col-0 and *shm1-2* at 0, 0.5%, and 2% sucrose conditions. Data presented were means ± SEM, n = 3 experiments. Different letters above error bars indicated significant difference at $P < 0.05$, using two-way ANOVA with Tukey's test.

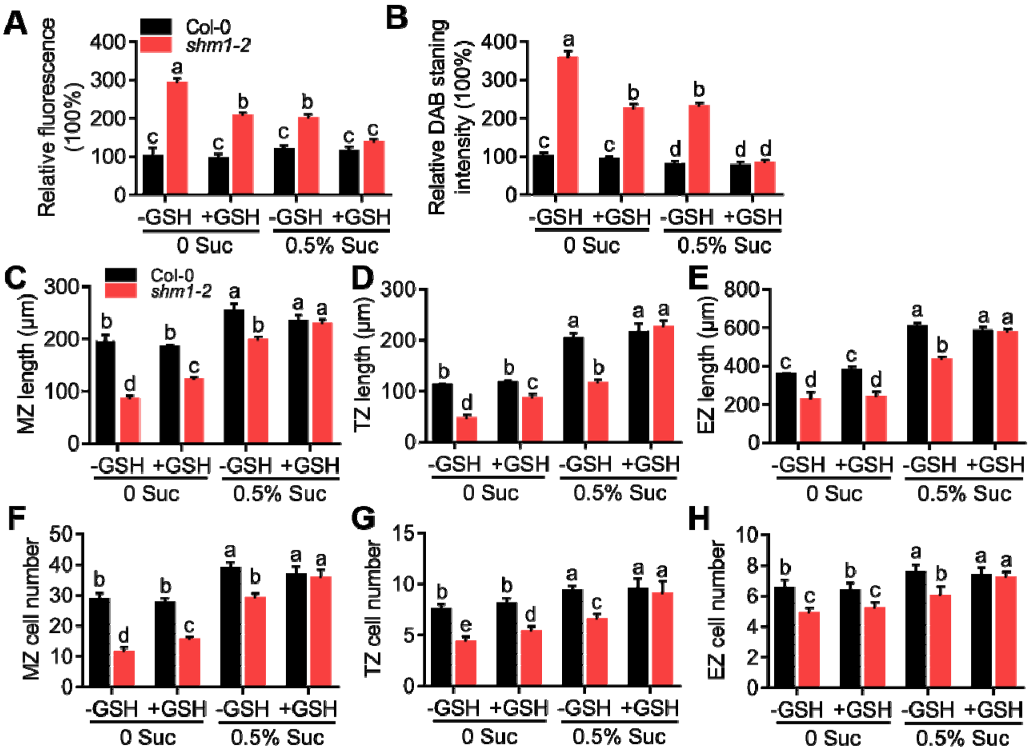

Supplemental Figure S6. GSH treatment partially rescued the reduced root meristem activity in the *shm1-2* mutant at low sucrose conditions. (**A**, **B**) Fluorescence intensity (**A**) and DAB staining intensity (**B**) in Col-0 and *shm1-2* by ImageJ in (**Figureure 6C**, **D**), respectively. The intensity in Col-0 at sucrose-free conditions was taken as 100%. In (**A**, **B**), data presented were means ± SEM, n = 3 experiments, each genotype with 10 roots per experiment. (**C**-**E**) The MZ, TZ, and EZ lengths of Col-0 and *shm1-2* treated with 0 or 0.5% sucrose plus 200 μM GSH or not for 8 days. (**F**-**G**) The corresponding cell numbers in the MZ, TZ, and EZ treated with 0 and 0.5% sucrose plus 200 μM GSH or not. Different letters above error bars indicated significant difference at P <0.05, using two-way ANOVA with Tukey's test.

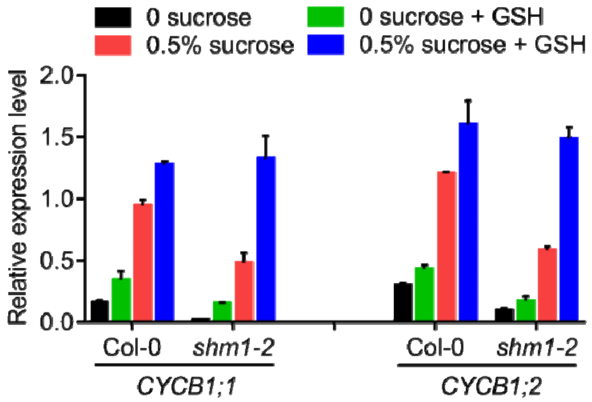

Supplemental Figure S7. GSH treatment partially attenuated the inhibition of cell cycle genes in the *shm1-2* mutant at low sucrose conditions. Quantitative PCR analyses of cell cycle marker genes (*CYCB1;1* and *CYCB1;2*) in Col-0 and *shm1-2* seedlings grown on 0 and 0.5% sucrose containing 1/2 MS medium with or without GSH for 8 days. Data presented were means ± SEM, n = 3. *EF1α* was used as an internal control.

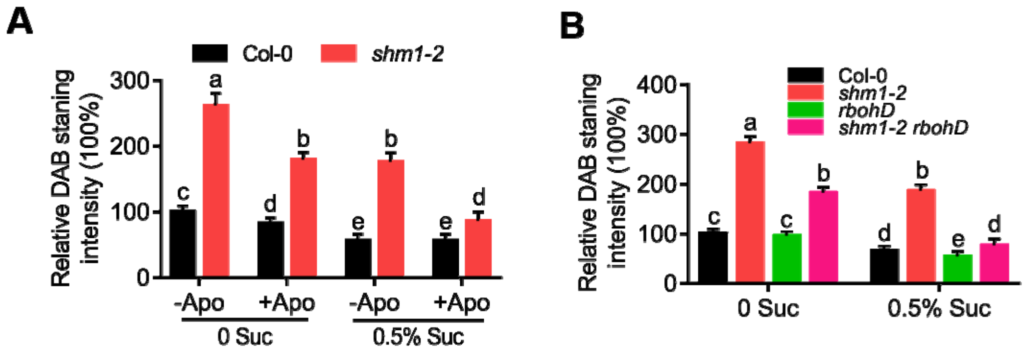

Supplemental Figure S8. ROS staining intensity in *shm1-2* and *shm1-2 rbohD* roots at low sucrose conditions. (**A**) DAB staining intensity in Col-0 and *shm1-2* with 10μM apocynin (the NADPH oxidase inhibitor) treatment or not at low sucrose conditions were determined by ImageJ in (**Figure 6C**). (**B**) DAB staining intensity in Col-0, *shm1-2*, *rbohD* and *shm1-2 rbohD* at low sucrose conditions were determined by ImageJ in (**Figure 6F**). Different letters above error bars indicated significant difference at P <0.05, using two-way ANOVA with Tukey's test. Data presented were means ±SEM, n = 3, each with 10 roots per experiment.

Table S1. Primers used in this study.

Gene	Gene reference	Use	Forward primer	Reverse primer
SHMT1	AT4G37930	*shm1-2* genotyping	TGGATTTGCGGTTTCTGTAAC	AGAAATGACCACAGCTTGTGG
SHMT1	AT4G37930	qRT-PCR	TACTCCTGCTCTCACTTC	CCTTCAACTTGGTTCCTT
SHMT1	AT4G37930	RT-PCR	ATGGCGATGGCCATGGCTCT	GTTCTTGTACTTCATGGTTTC
SHMT1	AT4G37930	Complement-ation	CGGGGTACCCCCGCATCAGAAGTCAAGGTAT	CCGGGATCCACAGAAAACACTTGTGGGGTGA
SHMT1	AT4G37930	Overexpression	GGGGACAAGTTTGTACAAAAAAGCAGGCTCCCCAAAAGCAGAGGCCACAA	GGGGACCACTTTGTACAAGAAAGCTGGGCCGTTCTTGTACTTCATGGTTTCTT
EF1α	AT5G60390	qRT-PCR	TGAGCACGCTCTTCTTGCTTTCA	GGTGGTGGCATCCATCTTGTTACA
ACTIN7	AT5G09810	RT-PCR	GGCCGATGGTGAGGATATTCAGCCACTTG	TCGATGGACCTGACTCATCGTACTCACTC
CYCB1;1	AT4G37490	qRT-PCR	GGAGGATAATCTCAAAAAACC	TCGAGCAGCAACTAAACCAAG
CYCB1;2	AT5G06150	qRT-PCR	TACATTGCAGTTCCACACCGGCTA	TAGCAACACCTCCATTCTCTGCCT
CYCB3;1	AT1G02930	qRT-PCR	TCATCCTTCGCAACCCC	GCTATGATCGCCATGTCCTT
CAT1	AT1G20630	qRT-PCR	CACCACAACAATCACCAT	TCCAGAGCAGACAATAGG
CAT2	AT4G35090	qRT-PCR	TGAACAAGAACATTGACAAC	CTGAGTATCGGCATAGGA
CAT3	AT1G20620	qRT-PCR	GAGATGAGGAGATCAATTACTACC	AACCTGTCTTGCCTGTCT
RBOHD	AT5G47910	qRT-PCR	GTACACCCACCATTTGTTCATC	AAAGCACGGAGCAGCCT
RBOHF	AT1G64060	qRT-PCR	GACACGCCAAGACGAAAGA	ACACCCCGTTGGTCAAGTT
SOD1	AT3G10920	qRT-PCR	GAACCTTGCTCCTTCCAGTG	TCTTCAGTTCTTTGTCTAGTCCG
RBOHD	AT5G47910	*rbohD* genotyping	GATGGGAGACAGCAGGAT	CGGATATGTACGCTCAGGT
